# Exploring Strategies to Optimise the Impact of Food-Specific Inhibition Training on Children’s Food Choices

**DOI:** 10.3389/fpsyg.2021.653610

**Published:** 2021-05-13

**Authors:** Lucy Porter, Fiona B. Gillison, Kim A. Wright, Frederick Verbruggen, Natalia S. Lawrence

**Affiliations:** ^1^School of Psychology, University of Exeter, Exeter, United Kingdom; ^2^Department for Health, University of Bath, Bath, United Kingdom; ^3^Department of Experimental Psychology, Ghent University, Ghent, Belgium

**Keywords:** inhibitory control training, response inhibition, food choice, childhood obesity, behavior change, digital interventions

## Abstract

Food-specific inhibition training (FSIT) is a computerised task requiring response inhibition to energy-dense foods within a reaction-time game. Previous work indicates that FSIT can increase the number of healthy foods (relative to energy-dense foods) children choose, and decrease calories consumed from sweets and chocolate. Across two studies, we explored the impact of FSIT variations (e.g., different response signals, different delivery modes) on children’s food choices within a time-limited hypothetical food-choice task. In Study 1, we varied the FSIT Go/No-Go signals to be emotive (happy vs. sad faces) or neutral (green vs. red signs). One-hundred-and-fifty-seven children were randomly allocated to emotive-FSIT, neutral-FSIT, or a non-food control task. Children participated in groups of 4–15. No significant FSIT effects were observed on food choices (all values of *p* > 0.160). Healthy-food choices decreased over time regardless of condition (*p* < 0.050). The non-significant effects could be explained by lower accuracy on energy-dense No-Go trials than in previous studies, possibly due to distraction in the group-testing environment. In Study 2, we compared computer-based FSIT (using emotive signals) and app-based FSIT (using neutral signals) against a non-food control with a different sample of 206 children, but this time children worked one-on-one with the experimenter. Children’s accuracy on energy-dense No-Go trials was higher in this study. Children in the FSIT-computer group chose significantly more healthy foods at post-training (*M* = 2.78, *SE* = 0.16) compared to the control group (*M* = 2.02, *SE* = 0.16, *p* = 0.001). The FSIT-app group did not differ from either of the other two groups (*M* = 2.42, *SE* = 0.16, both comparisons *p* > 0.050). Healthy choices decreased over time in the control group (*p* = 0.001) but did not change in the two FSIT groups (both *p* > 0.300) supporting previous evidence that FSIT may have a beneficial effect on children’s food choices. Ensuring that children perform FSIT with high accuracy (e.g., by using FSIT in quiet environments and avoiding group-testing) may be important for impacts on food choices though. Future research should continue to explore methods of optimising FSIT as a healthy-eating intervention for children.

## Introduction

The food we eat has a direct impact on our health (Afshin et al., 2019). A high intake of non-milk extrinsic sugars is associated with a high energy intake, and with long-term conditions such as obesity (Malik et al., 2013; Dong et al., 2015; Public Health England, 2015; SACN, 2015), Type 2 diabetes (Malik et al., 2010; Hu, 2013), and poor dental health (Sheiham and James, 2015; Meier et al., 2017). However, 98% of children in the United Kingdom consume more non-milk extrinsic sugar than the recommended limit (Public Health England, 2018), while only 18% meet the recommended five portions of fruit and vegetables per day (NHS Digital Lifestyles Team, 2019).

Given that the majority of children’s sugar intake comes from non-core foods such as soft drinks, biscuits, cakes, and puddings (Public Health England, 2015), replacing these sugary snacks with a piece of fruit could help to redress the existing dietary imbalance. However, early preferences for sweet vs. bitter flavours mean that children prefer energy-dense foods over fruit and vegetables (Birch and Fisher, 1998), with flavour often being the primary driver of children’s food choices (Nguyen et al., 2015). Younger children in particular are less likely to choose healthier foods over more palatable, energy-dense options (Ha et al., 2016). Energy-dense foods are often easily accessible, convenient, and highly visible (e.g., through marketing; Swinburn et al., 2011), and children are especially susceptible to the influence of food marketing (Boyland et al., 2016). Some strategies to encourage fruit and vegetable intake can also result in unintended negative consequences; for example, telling children that healthy foods have instrumental value (e.g., carrots help you to see in the dark) can actually decrease perceptions of tastiness and the likelihood of subsequent intake (Maimaran and Fishbach, 2014).

Many interventions to improve the nutritional quality of children’s diets are not successful, whilst those that are tend to be resource-intensive, multi-component interventions (Knai et al., 2006; Bourke et al., 2014; Hendrie et al., 2017; Johnson et al., 2018; Hodder et al., 2020), which may not be feasible to implement in all settings or with limited budgets (Ward et al., 2017). Digital behaviour change interventions (DBCIs) can reduce the costs associated with delivering interventions (e.g., time, personnel, and financial), and facilitate accessibility where attending in-person services is difficult or expensive (Murray et al., 2005; Sallinen et al., 2013; Price et al., 2014; Hayes et al., 2017; Sorgente et al., 2017). DBCIs are also a prime platform for delivering content in a gamified way that appeals to children (Chow et al., 2020).

Food-specific inhibition training (FSIT) is an example of a DBCI that aims to gamify the learning of healthier eating habits. Users make motor responses (e.g., key presses or touchscreen taps) in response to stimuli presented on screen (typically healthy foods or neutral images), but refrain when energy-dense foods such as chocolate, sweets, and crisps are presented (Houben and Jansen, 2011; Lawrence et al., 2015). Playing this task leads to reduced intake and choice of energy-dense foods, both amongst adults (Jones et al., 2016; Aulbach et al., 2019) and children (Folkvord et al., 2016; Porter et al., 2018).

Food-specific inhibition training is an example of an intervention that targets “automatic” drivers of eating behaviour. Many health behaviour change interventions focus on education, and do not account for the influence of these “automatic” drivers of behaviour (Marteau et al., 2012; Johnson et al., 2018). However, these processes are crucial for eating behaviour; automatic reward responses to food predict craving and food intake (Lawrence et al., 2012; Boswell and Kober, 2016), particularly when inhibitory control is low, as is likely the case for children given that neural substrates associated with self-control are not mature until early adulthood (Bunge et al., 2002; Keller and Bruce, 2018). It was originally thought that FSIT impacted eating behaviour by strengthening response inhibition in the face of tempting stimuli, however, research with adult participants has found that FSIT effects are more likely to be driven by reductions in the reward appeal (devaluation) of foods paired with response inhibition (Veling et al., 2017b).

Devaluation of food stimuli also occurs after evaluative conditioning, whereby food stimuli are repeatedly paired with images that evoke some kind of emotive or evaluative response (e.g., positive and negative facial expressions), subsequently impacting liking and choice of those items (Hensels and Baines, 2016; Shaw et al., 2016). While it could be argued that FSIT may be a form of evaluative conditioning (i.e., the No-Go cue or the act of not responding could serve as a negative stimulus, leading to devaluation after repeated pairing with certain food stimuli), research has found that devaluation after FSIT results from response inhibition itself rather than evaluative conditioning (Chen et al., 2016).

If both FSIT and evaluative conditioning lead to devaluation of foods and subsequent behaviour change *via* different mechanisms, combining them into one task could have a cumulative impact on food choices. Our past research with children used a version of FSIT containing happy and sad emoji faces as the Go and No-Go signals, respectively (Porter et al., 2018) meaning that this “emotive-FSIT” version of the task arguably also contained an evaluative conditioning element. Whilst FSIT can also reduce children’s calorie intake when neutral response signals (e.g., different shapes) are used (Folkvord et al., 2016), it is unknown whether emotive signals can augment FSIT effects. This question is of particular interest given that our team has developed a free FSIT app (“FoodT”^1^) for iOS and Android devices, which uses neutral response signals (green and red circles). This app was developed based on FSIT validated in adults (e.g., Lawrence et al., 2015, 2018) and has not yet been tested with children. If emotive signals are found to be more impactful for child samples, such amendments could be easily implemented into future FSIT paradigms. To explore this, we ran a series of studies to investigate whether this ready-to-use FSIT app (which uses neutral response signals) and the computer-based FSIT used in earlier research (which uses emotive signals) yielded meaningfully different results in FSIT effects on children’s food choices.

## Study 1

Our first study tested whether combining FSIT and evaluative conditioning could enhance healthy-food choices (vs. standard FSIT). We used the same emotive-signal, computer-based task as in Porter et al. (2018) and developed a near-identical version (still computer-based) using neutral signals.^2^

We also aimed to explore whether FSIT effects endure beyond the period immediately post-training. Previous work has tested children’s eating behaviour within a single experimental session (Folkvord et al., 2016; Porter et al., 2018), whereas research with adults has found evidence of lasting effects of repeated FSIT sessions (e.g., four or more in a single week) on outcomes over a number of months (Lawrence et al., 2015). In this study, we aimed to investigate whether any FSIT effects on food choices would still be present 1 week later and whether these could be augmented or reinstated with a second FSIT “top-up” session.

Our primary research question was whether combining FSIT with evaluative conditioning (by using emotive response signals) leads to larger training effects (vs. control) compared to FSIT using neutral signals. We hypothesised that children who completed FSIT (emotive or neutral) would choose a greater number of healthy foods in a time-limited, hypothetical choice task than children who completed a control task. Secondary questions included whether FSIT effects on food choice would endure 1 week later, and whether a second top-up FSIT session would augment/reinstate any training effects 1 week later. Ethical approval for this study was granted by the University of Exeter CLES Psychology Ethics Committee (reference 2017/1638).

### Materials and Methods

#### Participants and Design

Participants for this study were children at two schools in the Exeter and East Devon (United Kingdom) areas, whose parents returned the participation consent form. School A was located in a ward where 94.7% of residents are White, 2.8% Asian, 0.4% Black, and the remainder of Mixed or Other ethnic groups. In 2020, the proportion of children eligible for free school meals (FSM) was 9.6% (national average 17.3%; ONS, 2020). School B was located in a ward where 98.8% of residents were White, 0.3% Asian, 0.1% Black, and the remainder of Mixed or Other ethnic groups. In 2020, the proportion of children eligible for FSM was 1.6% (school information collected *via* national and local government websites^3^).

Power calculations were conducted using G*Power 3.1 to find the required sample size to detect an effect size (*f*) of 0.3587 (taken from Study 2 of Porter et al., 2018) at 80% power for a study design with three conditions, three measurement points, and an alpha level of 0.05, yielding a target of 54 participants. This was increased to 90 participants (30 per condition) to insure against attrition over study sessions.

The study had a mixed design, with a between-subjects factor with three levels (FSIT-Emotive vs. FSIT-Neutral vs. Control) and a repeated-measures element (outcomes were measured immediately post-training in session 1, at the start of session 2, and immediately post-training in session 2).

#### Measures and Materials

##### Go/No-Go Training Task

This task was programmed using EPrime software and accessed on university-owned laptop computers. Stimuli appeared on the screen one at a time for 1,250 ms, followed by a 1,250 ms inter-trial interval. Participants were required to press the spacebar when the stimulus appeared with a Go-signal but not when the stimulus appeared with a No-Go-signal. In Session 1, the tasks consisted of five blocks of 32 stimuli, while in Session 2, a top-up session of three blocks was used. Accuracy (presented as correct trials out of 32) and reaction time (RT; presented as average response time in milliseconds) feedback was presented after each block.

Active FSIT stimuli were 16 food images identical to those used in earlier research (Study 2, Porter et al., 2018; eight healthy such as apples, blueberries, etc., and eight energy-dense such as chocolate, crisps), while Control-task stimuli were 16 games-equipment images (eight sports, eight technology). Stimuli were presented twice per block. In the FSIT-Emotive task, Go-signals were happy-face emojis and No-Go-signals were sad-face emojis. In the FSIT-Neutral and Control tasks, Go-signals were green “Go” signs and No-Go-signals were red “Stop” signs. Each stimulus was presented with two variants of the relevant signal type to encourage stimulus-response learning over stimulus-signal learning (Best et al., 2016; Bowditch et al., 2016). There were three variations of each signal type (i.e., three of each of Emotive-Go, Emotive-No-Go, Neutral-Go, and Neutral-No-Go).

##### Hypothetical Food-Choice Task

Food choices were measured immediately post-training in Session 1, at the start of Session 2, and immediately post-training in Session 2. This task was hosted on a university server and accessed *via* the web browser. About 16 food images (eight healthy, eight energy-dense) were presented on the screen in a grid. Six of the healthy-food images and six of the energy-dense food images were different images of the same food types shown in the active FSIT tasks (e.g., apple, chocolate bar), with the rest being novel foods that did not appear in the FSIT tasks. Some images were those used by Porter et al. (2018), with extra image sets being created with photos found online or photographed by the first author. Images presented approximately one portion of food. Three image sets were created so that different images could be shown at each of the three measurement points (these were was counterbalanced across participants).

Children clicked on the eight foods they wanted most within a 60-s time limit. A time limit was imposed based on findings that FSIT effects disappear when longer time-periods are allowed for deliberation (Veling et al., 2017a). If children did not select eight foods within the time limit, the researcher offered them a second attempt. The number of healthy-foods chosen was recorded as the outcome variable (as children were only allowed to choose eight foods, this was directly proportional to the number of energy-dense foods chosen). Children were asked to pretend that these were real foods they could eat, to motivate ecologically valid choices.^4^ Children were able to modify their choices as many times as they wanted to within the time limit.

#### Procedure

Letters were sent to parents, containing a brief study description and a consent form. Only children whose parents consented were invited to participate. Children took part in groups of 4–15 at a time. Group sizes were dependent on the requirements of the schools. Groups were mixed with regards to FSIT condition.

For session 1, groups of children were taken from the classroom to an activity area where the laptops were set up. Instruction sheets showed the specific response signals children should attend to (i.e., happy/sad faces or Go/Stop signs) and the experimenter delivered verbal instructions to aid understanding. Once children had been instructed to begin, the experimenter observed children’s performance to ensure they understood the task and provided additional instructions and support for children who were struggling with the task. As each child reached the end of the Go/No-Go task, the experimenter opened the instruction page for the first food-choice task (Food Choice 1) for each child and asked them to wait at the instruction screen (no foods visible). When all children were ready, the experimenter again delivered verbal instructions to accompany those present on screen, emphasising the time limit and that they should pretend that they were choosing real foods to eat.

After a week-long interval, Session 2 began with a food-choice task (Food Choice 2a) followed by a “top-up” of the same Go/No-Go training task as before, and then a final food-choice task (Food Choice 2b). Before each task, children were given brief verbal instructions to refresh their memory.

#### Data Preparation and Analysis

Planned exclusion criteria included overall accuracy on the Go/No-Go task below 60%, No-Go accuracy below 50%, and average RTs beyond three SDs of the mean for that condition. Additional exclusions were made when Go/No-Go data were lost due to technical errors, researcher errors caused a deviation from the planned procedure (these included accidentally failing to counterbalance food choice image-sets, or presenting children with the wrong Go/No-Go task in the second session) and for child absence or requests to drop-out.

Repeated-measures ANOVAs investigated the effect of Condition on Go trial RTs, Go trial omission errors and No-Go trial commission errors across blocks. Models were analysed separately for each session. Where Mauchly’s test for sphericity was significant, corrections were used (Greenhouse-Geisser when epsilon <0.75, Huynh-Feldt otherwise). All pairwise comparisons were Bonferroni corrected.

An ANOVA was used to investigate the effect of Condition on the number of healthy-foods chosen in Food Choice 1. This analysis was one-tailed as it was a direct replication of the analyses conducted by Porter et al. (2018). Unadjusted planned comparisons between each FSIT group vs. the Control group were conducted (replicating earlier findings, as before). Bayes factors for these two planned comparisons were calculated using an online calculator (Dienes, 2014). For each comparison, the inputs to this calculator consisted of the mean difference between conditions, the standard error of this difference, and a prior based on all previous studies with children conducted by our research group and calculated using another calculator provided by Dienes and colleagues (prior = 0.8569); both the Bayes factor calculator and the prior calculator can be found online.^5^ A repeated-measures ANOVA investigated healthy-food choices across the three measurement-points. All analyses were conducted in SPSS v26. The full dataset is available at https://doi.org/10.24378/exe.3303.

### Results

#### Preliminary Analyses

Before exclusions, 112 children (59 female) aged 5–10 years (*M* = 7.93, *SD* = 1.84; age and gender information were missing for two children) were enrolled. Eight children were excluded from session 1 (absence on experiment days = 5, drop-out = 2, data loss = 1), with no further exclusions made on the basis of poor Go/No-Go task performance, resulting in a sample of 104 children (57 female) aged 5–10 years (*M* = 7.93, *SD* = 1.83). A further 11 children were excluded from session 2 (absence on experiment days = 6, experimenter error = 3, low Go/No-Go task accuracy = 2), resulting in a sample of 93 children (52 female) aged 5–10 years (*M* = 7.73, *SD* = 1.81) for these analyses. The minimum target sample size of 30 per condition was met in both sessions (see Table 1 below).

**Table 1:** Sample characteristics for each condition at each session.

	FSIT-emotive	FSIT-neutral	Control
Session 1 – n	34	35	35
Age – M (SD)	8.04 (1.88)	7.96 (1.81)	7.79 (1.86)
Gender – % female	52.9%	60.0%	51.4%
Session 2 – n	30	32	31
Age – M (SD)	7.82 (1.88)	7.78 (1.79)	7.60 (1.83)
Gender – % female	53.3%	62.5%	51.6%

One participant had missing data for Go RTs in the first block of Session 1 due to not making any correct Go responses in this block (the participant completed the task with 100% Go accuracy for the remaining blocks, meaning that they passed the accuracy inclusion criteria). This missing value was filled in with the mean for the participant’s age group and condition at Block 1, Session 1.

#### Go/No-Go Task Performance Analyses

**Figure 1:**
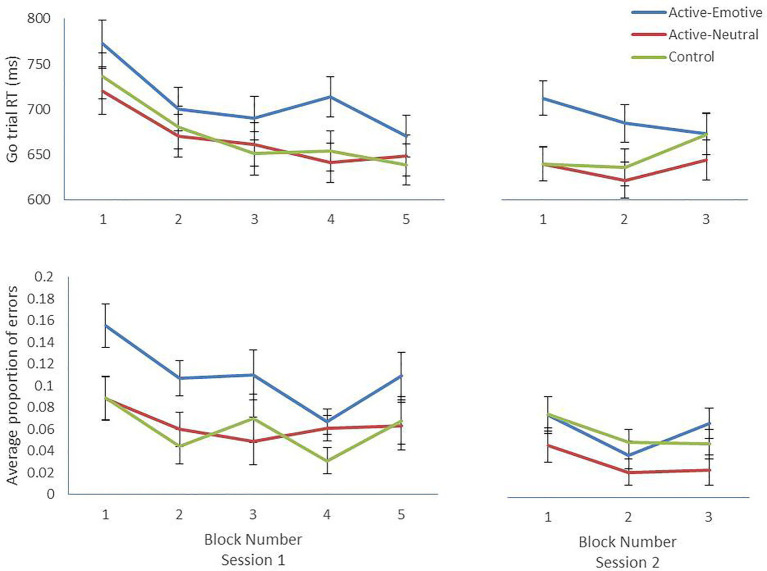
Mean and SE per block for Go trial Reaction Times and proportion of No-Go trial commission errors for each condition across blocks. Lower RTs/error rates indicate better performance.

In Session 1, RTs got significantly faster across blocks (*F_3.538, 357.341_* = 27.98, *p* < 0.001, *n*^2^_p_ = 0.217; Huynh-Feldt corrected; Figure 1), with no significant differences between conditions (*p* = 0.297). In Session 2, the Block × Condition interaction was significant (*F_4,180_* = 3.64, *p* = 0.007, *n*^2^_p_ = 0.075; Figure 1), with RTs getting faster over time in the Active-Emotive group (*F_2,89_* = 3.51, *p* = 0.034, *n*^2^_p_ = 0.073), getting slower in the Control group (*F_2,89_* = 4.30, *p* = 0.017, *n*^2^_p_ = 0.088) and remaining stable in the Active-Neutral group (*p* = 0.146).

Commission error rates improved significantly across blocks in Session 1 (*F_3.231,326.328_* = 4.48, *p* = 0.003, *n*^2^_p_ = 0.042; Huynh-Feldt corrected). Unexpectedly, there was a main effect of Condition (*F_2,101_* = 5.67, *p* = 0.005, *n*^2^_p_ = 0.101) with the FSIT-Emotive group showing significantly higher error rates (*M* = 0.109, *SE* = 0.012) compared to the FSIT-Neutral (*M* = 0.064, *SE* = 0.011, *p* = 0.019) and Control groups (*M* = 0.060, *SE* = 0.011, *p* = 0.009; Figure 1). In Session 2, commission error rates varied significantly across blocks (*F_2,180_* = 3.93, *p* = 0.021, *n*^2^_p_ = 0.042). There was a significant effect of Condition (*F_2,90_* = 3.10, *p* = 0.050, *n*^2^_p_ = 0.064), however, no pairwise-comparisons were significant.

#### Food Choices

The main effect of Condition was not significant, and healthy-food choices did not significantly differ between children in the FSIT-Emotive (*M* = 3.77, *SE* = 0.35), FSIT-Neutral (*M* = 3.91, *SE* = 0.36), and Control groups (*M* = 3.27, *SE* = 0.36) at Food Choice 1 (immediately after the first training; all values of *p* > 0.210). Bayes factors for the pairwise-comparisons sat between 1/3 and 3 (FSIT-Emotive BF = 1.15, FSIT-Neutral BF = 1.80), meaning that the evidence was not sufficiently conclusive to support either the null or alternative hypothesis.

**Figure 2:**
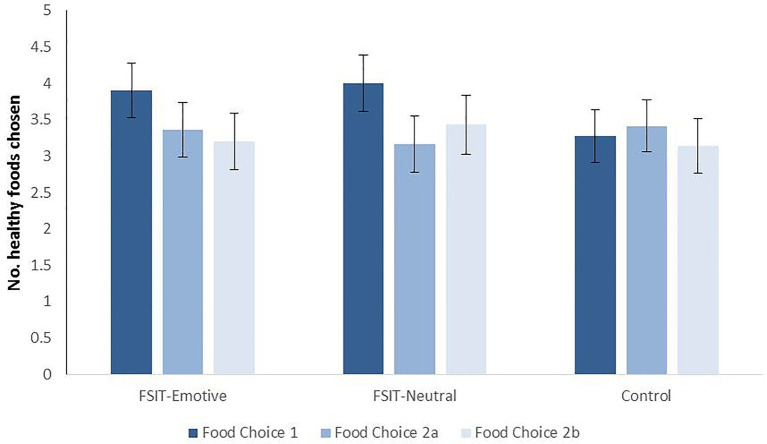
Mean number of healthy-foods chosen at each time-point for each condition, with SE. Food Choice 1 occurred immediately post-training in Session 1, 2a occurred 1 week later before the top-up training and 2b occurred immediately after the top-up training.

Healthy-food choices decreased significantly over time (*F_1.702,144.639_* = 3.29, *p* = 0.048, *n*^2^_p_ = 0.037; HF corrected; Linear Contrast *F_1,85_* = 4.42, *p* = 0.038; see Figure 2). Neither the effect of Condition nor the Time × Condition interaction was significant. Missing values were deleted listwise, meaning that different mean values for Food Choice 1 are presented in Figure 2 compared to those reported above, due to session 2 exclusions.

### Discussion

This study aimed to investigate whether combining evaluative conditioning and FSIT would encourage healthier choices among children compared to standard FSIT alone. We compared a task that used happy and sad faces as Go and No-Go signals, respectively (FSIT-Emotive condition) and a task that used neutral (green Go and red No-Go) signals (FSIT-Neutral condition) against a non-food Control task, measuring children’s food choices in a time-limited, hypothetical choice task at three time points. Our hypothesis of higher healthy-food choice in the FSIT tasks vs. Control was not confirmed; unexpectedly, we failed to replicate the significant training effects previously observed (Porter et al., 2018), despite the FSIT-Emotive task being identical to that used in the earlier research. Instead, there were no significant differences between groups at any time-point, and healthy-food choices decreased significantly over time with no evidence of this trend differing between groups.

Due to the non-significant results of this study, we were unable to determine whether evaluative conditioning can enhance FSIT effects on food choices. There are a number of differences between this study and the earlier study by Porter et al. (2018) that could help to explain the discrepancy in results. Firstly, in the earlier study, children were told that they would receive one of their food choices at the end of the day, to motivate ecologically valid choices. This was not possible in the present study for practical reasons. Children were encouraged to imagine that these were real foods that they would eat, but this may not have been enough, and future studies should aim to use real food outcomes to ensure ecological validity.

In addition, the food-choice tasks in the present study were timed by the computer and although children were not alerted to this feature, they were able to modify their choices as many times as they wanted to within the 60-s window. Comparatively, the earlier study involved researchers working one-on-one with children for this task, meaning that children could be prevented from changing their choices or deliberating for too long. Past research with a similar response training task has found that effects on food choices are eliminated when adult participants are given more time to make their choices (Veling et al., 2017a). These results could indicate that a similar effect occurs with children. Future studies should explore whether FSIT effects on food choices are impacted by the amount of time permitted for food choices.

Alternatively, it could be that group-testing in this study impacted children’s attention and engagement with the FSIT task (e.g., due to distraction by other children). The FSIT-Emotive task had a significantly higher no-go commission error rate than the other two tasks, with a mean of 0.109. The mean commission error rate for the same task in the earlier study was 0.063 (where children were tested individually, or in smaller groups of a maximum of four with two researchers present; Porter et al., 2018). A meta-analysis of studies with adult participants found that accuracy on inhibition trials is a crucial predictor of training effects on outcomes (Jones et al., 2016). Therefore, poorer task performance in the current study may have minimised training effects and resulted in the non-significant effects observed here. The FSIT-emotive task may have been impacted more than the other tasks due to the highly-similar Go and No-Go signals (i.e., yellow circles with small variations in facial expression, compared to potentially more easily-discriminable green and red signs). Future studies should ensure that children can concentrate and engage with the FSIT task.

## Study 2

In Study 2, we implemented the methodological recommendations of Study 1 (i.e., using real food rewards to improve ecological validity of outcome measures; implementing FSIT individually in a quieter, less distracting environment) to compare the FSIT-emotive task against the neutral FSIT task included in the FoodT app. Children worked with the experimenter one-on-one to create a more controlled testing environment, and when taking part in the time-limited hypothetical food-choice task, children were told that they would receive one of their choices at the end of the study. Real-food choices were also measured. Thirdly, a baseline measure of hypothetical food choices was taken to help understand (i) whether groups were well matched in their healthy-food choices at the outset, and (ii) whether any changes occurred within groups from pre to post-training. Finally, the hypothetical food-choice task was changed to a card-based game (as in Porter et al., 2018), rather than the computer-based task used in Study 1. These methodological changes brought the method of Study 2 more closely in line with the methods used in Porter et al. (2018).

As described earlier, FoodT is a FSIT app that uses neutral response signals (red and green circles, similar to the colour-based signals of the FSIT-neutral task of Study 1) that was developed based on FSIT tasks that had been validated in adult samples (e.g., Lawrence et al., 2015). Preliminary work with adults using FoodT at home has revealed reduced self-reported snacking and greater self-reported weight loss, although the effect is smaller than that observed with web-based training accessed *via* laptop or desktop computers (Lawrence et al., 2018). FoodT has not yet been tested for its efficacy at changing children’s eating behaviours. We decided to test this app directly (rather than reusing the FSIT-neutral task in Study 1) as FoodT is a ready-to-use app that could be delivered immediately to families with children if there is evidence of its effectiveness. Unpublished feasibility studies conducted by our research group have shown that families prefer touchscreen-compatible tasks, which accords with wider trends showing increases in children’s use of touchscreen devices such as tablets (Ofcom, 2020). While it would not be possible to isolate the effects of emotive vs. neutral signals alone due to other differential features between the two tasks (e.g., touchscreen vs. keyboard response, the use of “filler” stimuli in FoodT, clearer point scoring system in FoodT; see Table 2 below), it would at least be possible to understand whether FoodT produces comparable results to the computer-based task tested successfully in earlier research (Porter et al., 2018). If not, this would indicate that further development and optimisation of the app may be needed.

**Table 2:** Differences between the food-specific inhibition training (FSIT)-computer and FSIT-app tasks.

	FSIT app	FSIT computer	Control
Delivery mode	iPad (FoodT)	Laptop (EPrime)	Laptop (EPrime)
Number of blocks	6	5	5
Trials per block	32	32	32
Critical trials per block	16	32	0
Trial length (inter-trial interval)	1,500 ms (500 ms)	1,250 ms (1,000 ms)	1,250 ms (1,000 ms)
Go trial stimuli	Healthy food (e.g., fruit)	Healthy food (e.g., fruit)	Sports-equipment (e.g., goggles, balls)
No-Go trial stimuli	Energy-dense food (e.g., chocolate, crisps)	Energy-dense food (e.g., chocolate, crisps)	Technology (e.g., TVs, games consoles)
Filler stimuli	Yes (clothes, flowers, stationery)	No	No
Response signals	Green vs. red circles	Happy vs. sad emoticons	Happy vs. sad emoticons
Signal delay	Yes (100 ms)	None	None
Feedback	Trial-by-trial point scoring presented; End of block feedback Accuracy: % Speed: milliseconds	End of block feedback only; Accuracy: score/32 Speed: seconds	End of block feedback only; Accuracy: score/32 Speed: seconds

An additional aim was to pilot a measure of food liking that could be used to investigate whether food devaluation occurs after children complete FSIT. No research has yet investigated the mechanisms of FSIT with children, and this study aimed to make the first steps towards testing the devaluation hypothesis (Veling et al., 2017b) with this population. A further outcome measure tested here was whether children’s first choice in the hypothetical food-choice task was more likely to be a healthy food after FSIT compared to control.

Our primary research question was whether the computer-based FSIT task used in our earlier studies (Porter et al., 2018) leads to a larger training effect (vs. control) compared to app-based FSIT. We hypothesised that children who completed FSIT (computer or app) would choose a greater number of healthy foods in a time-limited, hypothetical food-choice task than children who completed a control task. Our secondary research questions were (i) whether children who completed FSIT (computer or app) would rate their liking for energy-dense foods as lower compared to children in the control group, and (ii) whether children would be more likely to choose a healthy food as their first choice in the time-limited hypothetical food-choice task. This study was pre-registered at https://osf.io/2v7hg/. Ethical approval was granted by the University of Exeter CLES Psychology Ethics Committee (reference eCLESPsy000031 v4.1).

### Materials and Methods

#### Participants and Design

This study had a mixed design with a three-level between-subjects factor (FSIT-app vs. FSIT-computer vs. Control) and a within-subjects repeated outcome assessment. Two outcome measures were assessed at baseline and post-training (the number of healthy foods chosen in the hypothetical food-choice task, and food-liking ratings), while real-food choice was measured at the end of the study only.

A power analysis conducted using G*Power 3.1.9.2 revealed that a sample of 192 participants would be required to achieve 80% power with an alpha level of 0.05 and a medium effect size (*f* = 0.25).^6^ As the main hypothesis involved comparing each FSIT group to the Control group, the power analysis was conducted for an ANCOVA with two groups and one covariate, with the resulting sample size (*n* = 128) then being multiplied by 1.5 to achieve the correct sample size for a design with two FSIT groups to be compared against a Control group (*n* = 192).

Three primary schools in London were approached to participate in the study, with all three responding and consenting. School A had 9.2% of pupils eligible for FSM (national average = 17.3%; ONS, 2020), and was located in the borough of Brent, where in 2018 32.6% of residents were Asian, 31.1% were White, 18.9% were Black and the remainder were of Mixed or Other ethnicity. School B had 15.6% of pupils eligible for FSM, and was located in the borough of Southwark where in 2018, 61.0% of residents were White, 19.5% were Black, 5.2% were Asian, and the remainder were of Mixed or Other ethnicity. School C had 27.8% of pupils eligible for FSM and was located in the borough of Lambeth, where 52.4% of residents were White, 23.2% were Black, 8.5% were Asian, and the remainder were of Mixed or Other ethnicity. Data on schools was obtained from national and local government websites.^7^

#### Measures and Materials

##### Go/No-Go Training Task

As in Study 1, all tasks consisted of stimuli appearing on screen, one-by-one, accompanied by a Go or a No-Go signal. The FSIT-Computer and Control tasks were both programmed using EPrime and delivered *via* laptop, and consisted of five blocks of 32 stimuli presentations as in earlier studies. The FSIT-app task was delivered on an Apple iPad and consisted of six blocks of 32 stimuli presentations (two separate games of FoodT, which consists of three blocks per game). This ensured roughly equivalent gameplay time (approximately 5 min) across conditions due to the slightly faster pace of the FSIT-app task.

The FSIT-computer task was adapted from Study 1 to contain the same eight healthy-food images (Go trials) and the same eight energy-dense food images (No-Go/trials) as the FSIT-app task. These images appeared twice per block in the FSIT-computer task (as in previous studies) but only once per block in the FSIT-app task as this task also presented participants with eight “filler” stimuli (i.e., flowers, clothing, and stationery), which were each presented twice per block, once as a Go stimulus and once as a No-Go stimulus. The Control task contained eight sports-equipment images (Go trials) and eight technology images (No-Go trials), all presented twice per block.

In the FSIT-app task, the Go signal was a green ring encircling the stimulus and the No-Go signal was a red ring encircling the stimulus. These rings appeared 100 ms after stimulus onset and remained on screen for the duration of the stimulus. In the FSIT-computer and Control tasks, the Go signal was a happy emoticon and the No-Go signal was a sad emoticon that appeared at the same time as the stimulus and remained on screen for the duration (as before, three different exemplars of each signal type were used in the two computer-based tasks, with each stimulus being presented with two variants to encourage Stimulus-Response learning over Stimulus-Signal learning; Best et al., 2016).

There were a number of further differences between the FSIT-app task and the two computer-based tasks; a summary of the differences between the tasks is presented below in Table 2. As noted in the introduction to this study, we chose specifically to compare the FSIT-app task against a version of FSIT that has previously been found to impact children’s food choices (e.g., see Porter et al., 2018). For this reason, and to maintain consistency with the task in Study 1, the FSIT-computer task was not reprogrammed to accommodate these differences.

##### Hypothetical Food-Choice Task

Following the methods of Porter et al. (2018), children were shown 12 food-image cards (six healthy, six energy-dense), of which they could choose six. Four of each food type were different exemplars of foods presented in training and two were novel, untrained foods. To motivate ecologically valid choices, children were informed that they should choose foods that they really wanted, as they would be getting one of these foods at the end of the experiment. They were also informed that they would be given 30 s to complete the task as research has shown that FSIT effects disappear when more time is given for deliberation over choices (Veling et al., 2017a). If children completed their choices within 30 s, the researcher ended the task, preventing any further changes to selections. The researcher informed children that time was running out as the 30 s limit approached.

Images were printed on paper, laminated, and cut into sets of cards. Two different image sets were developed which were counterbalanced among participants from pre- to post-training. The number of healthy foods chosen was the primary outcome measure. The first food that children chose was also recorded as a novel secondary outcome measure. Whilst the images included in the choice tasks were judged to be equally attractive across categories (i.e., healthy and energy-dense) by the research team, they were not systematically matched for palatability and attractiveness as no data currently exists regarding children’s ratings of food stimuli. However, the food rating task described below made a first attempt at piloting a measure to obtain this information from children.

##### Food-Liking Rating Task

**Figure 3:**
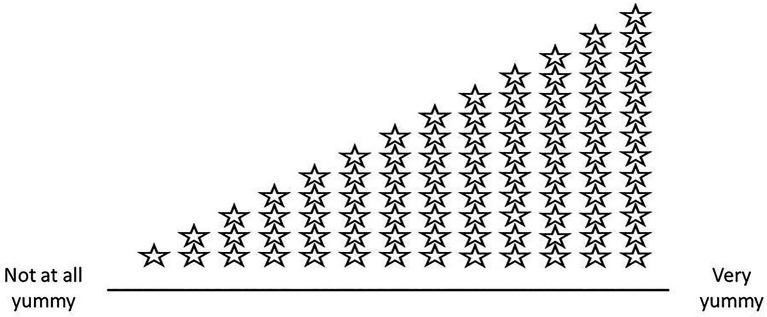
Visual analogue scale used to rate food-liking.

Children were shown 12 images of food (six healthy, six energy-dense), one at a time. Four of each food type were different exemplars of foods presented in training, whilst two were novel, untrained foods. Images in the liking rating task were different to those presented in the hypothetical food-choice task. Children were asked to rate each food on a 100-point visual analogue scale (VAS) ranging from “Not at all yummy” all the way up to “Very yummy”. The number ratings were not visible on the scale, but a visual aid was available in the form of increasing numbers of stars above the line as it approached the “Very yummy” end (visually, this resembled a “wedge” made up of stars that hovered above the length of the line; see Figure 3).

Children were discouraged from counting the stars and were advised to use the visual aid as a rough guide to prevent them from remembering their rating for a given food from one session to the next (for the same reason, previously tested measures using a smaller number of categories to indicate liking were not appropriate for this study). Children pointed to the location on the line that they would rate the food, and the experimenter marked a line with a pen to show where the child’s finger had landed. Later, these marks were measured for their location along the line, and converted into a value out of 100. Images were printed on paper, laminated, and cut into sets of cards. The same images were rated at pre- and post-training. Again, whilst chosen images across categories were judged to be equally attractive by the research team, they were not systematically matched for palatability and attractiveness as no data currently exists regarding children’s ratings of food stimuli. However, this task makes a first attempt at piloting a measure to obtain this information from children.

##### Hunger Scale

The five-point hunger scale developed by (Bennett and Blissett, 2014) was used. This depicts a series of teddy bears with increasing amounts of “food” in their tummies, and ranges from “very hungry” to “very full”, with an option of “just right” in the middle. Hunger was measured at the start of the second session (i.e., the training session) only, as previous work has suggested that hunger levels may influence the efficacy of the training task (Veling et al., 2013). Lower scores indicated greater hunger, while higher scores indicated increasing fullness.

##### Real-Food-Choice Task

Children were offered a selection of snacks from which they could choose one to take home as a participation reward. The options included fruit (apple, orange, and small bunch of green grapes) and energy-dense snacks (medium-sized Kinder chocolate bar, Nairn’s gluten-free chocolate chip biscuits, and Walker’s baked crisps). An example of each food was placed on a paper plate, (the actual foods that children would be given were kept in staffroom refrigerators or in a cool bag) and these example options were kept covered by a tea towel until the real-food-choice task began. Children chose one option (this choice was noted as an outcome measure) and were subsequently also allowed an extra choice of one piece of fruit (to ensure all children went home with at least one piece of fruit). No time limit was imposed on this task. Children’s choices were placed in paper bags, stapled closed with a debrief letter for parents attached, and handed to teachers at the end of the day.

##### Debrief and Awareness Assessment

Children were asked a series of questions to assess their awareness of the aims of the project: (i) what they thought the games they had played were about, (ii) why they thought they had played them, (iii) if they could remember which pictures (Control) or foods (FSIT) they had to press during the computer/iPad game, and finally (iv) if they thought that the computer/iPad game might have changed which foods they wanted. Children’s answers were coded as aware/unaware for the following: (i) awareness of contingencies, (ii) awareness of healthy eating purpose, and (iii) awareness of task effects on food choices.

#### Procedure

Letters were sent home to parents, containing a brief description of the study, and a consent form. Only children whose parents consented to participation were invited to take part. All children worked with the researcher individually. In the first session, children were asked if they assented to playing a few quick games about their favourite foods. Children completed the baseline hypothetical food-choice task and food-liking rating task before returning to the classroom. Session 1 lasted for approximately 5 min.

The second session took place during the following school week. Children were again asked if they assented to participating. The second session began with the hunger rating scale, before the Go/No-Go training task. Children then completed the hypothetical food-choice task and the food-liking rating task. The order of these tasks remained fixed due to food choices being our primary outcome measure. Finally, the experimenter presented children with the real-food-choice task, and asked children to choose one item to take home as a thank you for taking part. After their choices had been made, children were asked the awareness questions and were debriefed before returning to the classroom.

#### Data Preparation and Analyses

Planned exclusion criteria included overall accuracy on the Go/No-Go task below 60%, No-Go accuracy below 50%, and average RTs beyond three SDs of the condition group mean.

To check whether the food pictures presented in the liking rating task were well matched, repeated-measures ANOVAs were conducted with a two (food type: healthy vs. energy-dense) by two (included in FSIT tasks vs. novel) design. This analysis was conducted as a preliminary check considering that, as noted above, stimuli were not systematically matched for palatability and attractiveness as no data currently exists regarding children’s ratings of food stimuli.

Repeated-measures ANOVAs were used to investigate reaction times on Go trials and No-Go commission errors across blocks. For the FSIT-app condition (for which six blocks of training were completed), only the first five blocks were entered into analyses so that comparisons could be made across conditions. Where the assumption of sphericity was violated, corrections were used (Greenhouse-Geisser where epsilon < 0.75, Huynh-Feldt otherwise). The data from the FSIT-app condition was also analysed in repeated-measures ANOVAS to see whether reaction times and error rates across blocks differed for food stimuli (which were presented with constant stimulus-response associations) vs. filler stimuli (50/50 stimulus-response associations). This allows us to differentiate between performance improvements based on general task practice vs. those based on learning specific stimulus-response (go or no-go) associations (e.g., Lawrence et al., 2015).

The effect of training group on hypothetical food choices was explored using an ANCOVA model, with baseline choices entered as a covariate and post-training choices as the outcome measure. Pairwise comparisons were conducted to investigate differences between the three groups (these were unadjusted as they replicated earlier findings). Bayes factors for each FSIT vs. Control comparison were calculated using the method and calculator described in Study 1. Paired samples *t*-tests were conducted for each condition separately to test the change in number of healthy foods chosen between the two measurement points. Binary logistic regression models were analysed to test whether children in the two FSIT groups (compared to the Control group) were more likely to choose (i) a healthy food as their first choice in the hypothetical food-choice task, and (ii) a healthy food as their real food participation reward.

Food-liking ratings were analysed with repeated-measures ANOVAs, including the within-subjects factors of food health status (healthy vs. energy-dense) and time (baseline vs. post-training), with condition as a between-subjects factor. We had also planned to include a within-subjects factor indicating whether foods had been included in the FSIT tasks (included vs. novel), however, baseline analyses indicated that included vs. novel foods were not well matched and could not therefore serve as an appropriate comparison (see below). All analyses were conducted in SPSS v26 and the dataset is available at https://doi.org/10.24378/exe.3303.

### Results

#### Preliminary Analyses

In total, 219 children (115 female) aged 4–10 years (*M* = 6.64, *SD* = 1.80) were randomised to the FSIT App (*n* = 72), FSIT Computer (*n* = 73), and Control (*n* = 74) groups. Thirteen were excluded due to either low Go/No-Go task performance accuracy (i.e., lower than 60%; *n* = 8) or absence from school during the second session (*n* = 5). The data from 206 children (106 female) aged 4–10 years (*M* = 6.77, *SD* = 1.76) were retained.

The three training groups (FSIT-app, FSIT-computer, and Control) were well balanced with regards to age, gender, baseline food choices, baseline ratings for each of the four food types (healthy trained, healthy novel, energy-dense trained, and energy-dense novel), and hunger during the training session (Table 3).

**Table 3:** Group demographic characteristics and baseline outcome measures.

	App (*n* = 70)	Computer (*n* = 69)	Control (*n* = 67)
Age	6.99 (1.80)	6.62 (1.71)	6.69 (1.79)
Gender – *n* female (%)	37 (52.9)	30 (43.5)	39 (58.21)
Healthy-food choices	2.54 (1.21)	2.87 (1.45)	2.57 (1.29)
Healthy trained rating	72.60 (18.41)	71.68 (20.62)	69.14 (21.30)
Healthy novel rating	58.68 (27.70)	54.71 (31.34)	57.44 (30.76)
Energy-dense trained rating	74.18 (19.11)	77.30 (18.42)	71.10 (21.79)
Energy-dense novel rating	79.72 (20.61)	77.88 (21.99)	75.11 (21.87)
Hunger	2.57 (1.27)	3.04 (1.39)	2.85 (1.47)

For gender, frequencies of female participants are noted with percentage of group in brackets. All other variables are described in terms of mean averages, with SDs in brackets.

#### Baseline Food Ratings

At baseline, a significant effect of health status was found (*F_1,203_* = 45.17, *p* < 0.001, *n*^2^_p_ = 0.182), with healthy foods being rated as liked less than energy-dense foods. Foods that were included in the training were liked more than the novel foods (*F_1,203_* = 21.19, *p* < 0.001, *n*^2^_p_ = 0.095), suggesting that the novel stimuli chosen in this study were not well matched (no exposure to the training task had occurred at this point). Due to these unintended baseline differences in liking for foods included in the training vs. novel foods, subsequent analyses only focused on those foods that had been included in the training, as the novel foods could not be used for comparison.

#### Training Performance

Reaction times got significantly quicker over blocks (*F_3.28,659.282_* = 42.03, *p* < 0.001, *n*^2^_p_ = 0.173). A significant effect of condition was found (*F_2,201_* = 34.29, *p* < 0.001, *n*^2^_p_ = 0.254) with slower RTs for participants in the FSIT-app condition (*M* = 884.93, *SE* = 16.83) compared to participants in both the FSIT-computer (*M* = 703.79, *SE* = 16.83, *p* < 0.001) and control (*M* = 726.19, *SE* = 17.20, *p* < 0.001) groups. A significant interaction between block and condition (*F_6.56,659.282_* = 3.08, *p* = 0.004, *n*^2^_p_ = 0.030) was also observed, with simple effects analyses revealing that improvements in RTs over blocks were strongest for the FSIT-app group (*F_4,198_* = 18.42, *p* < 0.001, *n*^2^_p_ = 0.271), followed by the FSIT-computer group (*F_4,198_* = 7.22, *p* < 0.001, *n*^2^_p_ = 0.127) and finally the control group (*F_4,198_* = 3.88, *p* = 0.005, *n*^2^_p_ = 0.073).

Commission errors decreased over blocks (*F_3.650,733.550_* = 11.426, *p* < 0.001, *n*^2^_p_ = 0.054), and a significant effect of condition (*F_2,200_* = 11.41, *p* < 0.001, *n*^2^_p_ = 0.100) revealed lower error rates in the FSIT-app group (*M* = 0.031, *SE* = 0.007) compared to the FSIT-computer (*M* = 0.067, *SE* = 0.007, *p* = 0.001) and control (*M* = 0.072, *SE* = 0.007, *p* < 0.001) groups. No significant interaction was observed for this analysis.

In analyses on FSIT-app data only, there was no evidence of an effect of Stimulus Type (food vs. filler) on RTs, nor was there evidence of an interaction between Stimulus Type and Block for RTs (both *p* < 0.200). Commission errors were significantly higher for filler stimuli (*M* = 0.055, *SE* = 0.007) than for energy-dense food stimuli (*M* = 0.028, *SE* = 0.005; *F_1,68_* = 33.22, *p* < 0.001, *n*^2^_p_ = 0.328), suggesting participants learned food-No-Go associations as expected. No interaction was found between block and stimulus type for commission errors.

#### Food Choices

Post-training healthy-food choices differed significantly between conditions (*F_2,202_* = 5.74, *p* = 0.004, *n*^2^_p_ = 0.054) with the highest healthy-food choice in the FSIT-computer group (*M* = 2.78, *SE* = 0.16) followed by the FSIT-app group (*M* = 2.42, *SE* = 0.16) and finally the control group (*M* = 2.02, *SE* = 0.16). Planned pairwise comparisons revealed that the only significant difference existed between the FSIT-computer group and the Control group (*p* = 0.001), with the comparison between the FSIT-app and Control groups failing to pass the significance threshold (*p* = 0.077). There was no significant difference between either of the two FSIT groups either (*p* = 0.103). Bayes factors show that the data indicates strong support for a difference between the control group and the FSIT-computer task (BF = 210.98) but that the data are inconclusive for the FSIT-app task (BF = 1.80).

**Figure 4:**
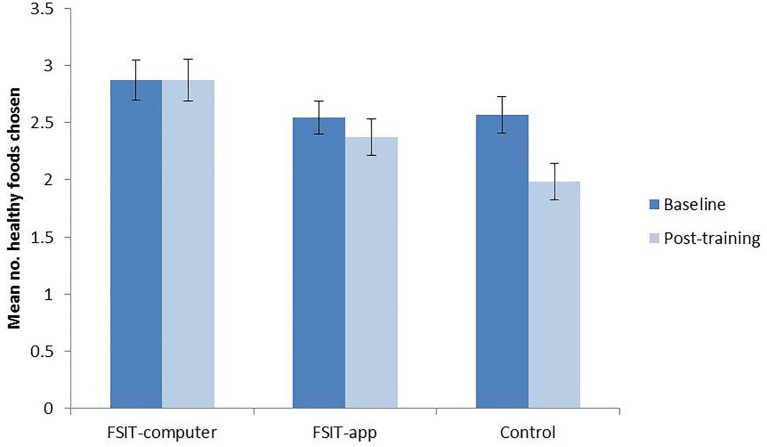
Mean number of healthy foods chosen at baseline and post-training within each condition; error bars show SE.

Paired sample *t*-tests revealed that the effect of condition was primarily driven by a decrease in healthy-food choice in the Control condition across time-points (Figure 4). Comparing baseline food choices to post-training food choices revealed no evidence of change in the FSIT-app (*p* = 0.334) or FSIT-computer (*p* = 1.000) groups, but a significant effect of time was found in the Control group (*t_66_* = 3.56, *p* = 0.001) with choices at post-training (*M* = 1.99, *SD* = 1.32) being significantly lower than those at baseline (*M* = 2.57, *SD* = 1.29).

Binary logistic regression revealed that compared to the Control group, participants in the FSIT-computer group were no more likely to select a healthy food as their first choice in the post-training hypothetical choice task (*p* = 0.052) and nor were those in the FSIT-app group (*p* = 0.653).

Across the entire sample, only 14.8% of children chose a healthy food in the real choice reward task and when examining the effect of condition on real-food choices, there was no significant effect of completing either the FSIT-app or FSIT-computer training compared to the Control task (both *p* > 0.400).

#### Food-Liking Ratings

**Figure 5:**
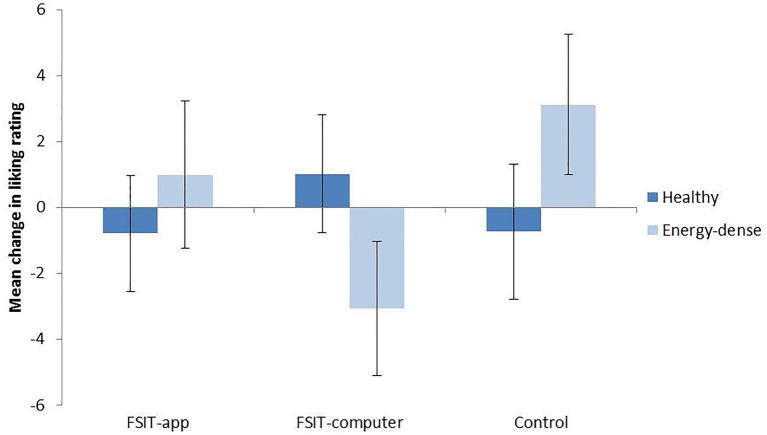
Mean change (plus SE) from baseline to post-training in food-liking ratings for healthy foods and energy-dense foods.

These analyses were conducted for trained foods only, due to the finding that trained foods and novel foods were not well matched at baseline. Healthy foods were rated slightly lower (*M* = 70.95, *SE* = 1.38) than energy-dense foods (*M* = 74.78, *SE* = 1.25, *F_1,197_* = 4.66, *p* = 0.032, *n*^2^_p_ = 0.023) but no further significant main effects or interactions were observed. For healthy foods, a slight decrease in liking was observed for the FSIT-app group and the Control group, whereas a slight increase was observed in the FSIT-computer group (Figure 5). The opposite patterns were observed for unhealthy items, with liking ratings decreasing slightly in the FSIT-computer group and increasing slightly in the FSIT-app and Control group. However, none of these differences or changes reached significance (all *p* > 0.130).

#### Effect of Awareness

One-hundred-and-eighty-six children in the sample were interviewed at the end of their involvement with the project (some children were not interviewed either due to time constraints or due to difficulties maintaining attention i.e., for very young children). The majority of children were aware of task contingencies (*n* = 152) but awareness of the healthy-eating aims of the study and task effects were much lower (*n* = 62 and 39, respectively). Chi-squared tests revealed that there were no significant differences between groups for any of the awareness measures (all *p* > 0.480). In addition, adding these variables to the ANCOVA investigating the effect of training on food choices revealed that none were predictive of food choices (all *p* > 0.290), while the effect of condition remained significant (*p* = 0.004).

### Discussion

In this study, we tested a FSIT-app against the FSIT-computer task, we have used in previous research (Porter et al., 2018). We hypothesised that children playing the two FSIT tasks (app or computer) would choose a greater number of healthy foods compared to children playing the Control task. We were also interested in whether there would be any preliminary evidence for differences in effect sizes of these respective FSIT tasks (when each was compared to the Control task). Our findings partially support our hypothesis; children in the FSIT-computer group chose a significantly greater number of healthy foods in the post-training hypothetical food-choice task. In addition, within-group analyses showed that healthy-food choices in the control group decreased over time, whereas they remained stable in the two FSIT groups. This suggests that FSIT can have a beneficial effect on healthy eating behaviours. Whilst there was a trend for children in the FSIT-app group to choose a greater number of healthy foods than children who had played the control task, this difference was not significant. The within-group analyses showed that the FSIT-app group also appeared to be protected from the decline in healthy-food choices observed in the Control group; however, the lack of significant differences at post-training means that no definitive conclusions can be drawn regarding the effects of this task on food choices.

There was no evidence that either of the FSIT tasks had any effect on real-food choices. Previous research has found that FSIT can impact children’s food choice and eating behaviours when faced with real foods; Folkvord et al. (2016) found that children who had played FSIT ate less than children who had played control training when they were given free access to sweets and chocolate, and Porter et al. (2018) found that children who had played FSIT chose a greater number of fruit items (relative to energy-dense foods) to go into their snack bags compared to children who had played control training. It is possible that the present non-significant effects are due to wash-out of training effects in the current study, as the real-food-choice task came at the very end of the experiment after the hypothetical food-choice task and the food-liking rating task. In addition, the real-food-choice task (in which children were allowed a single food choice) may not have been sensitive enough to detect differences between groups compared to those used by other studies (e.g., calorie intake in Folkvord et al., 2016 and a task where children were allowed three items in Porter et al., 2018). Thus, our real-food choice measure depended on training effects being of an “all or nothing” nature, whereas FSIT effects might be more subtle than this [e.g., the children who played FSIT in the study by Folkvord et al. (2016) consumed 34% fewer calories than their peers in the control group]. Children were also allowed more time to deliberate over their choices in this task than they were in the time-limited, hypothetical food-choice task. Work with adults has shown that the effects of response training paradigms can be highly dependent on impulsive choice contexts (Veling et al., 2017a), which provides another potential explanation for these non-significant effects.

A new measure of food devaluation for use with children was piloted in this study. Devaluation of foods associated with response inhibition has been observed in previous studies with adults (Veling et al., 2017b). On the whole, children were able to complete the task, indicating its suitability for use with younger samples. However, there were no significant differences between groups on change in liking ratings for either healthy or energy-dense foods. This may be because this study was powered to detect between-groups differences in food choices but not in children’s food ratings. It is also possible that using VAs with child participants is not a particularly sensitive method for assessing food devaluation; histograms of children’s food ratings revealed that some children were only selecting extreme values for their ratings of the food stimuli, which would preclude the detection of subtle changes in food-liking. Nevertheless, it is interesting that the means showed a subtle trend for devaluation in the FSIT-computer group only (which was also the only group to show significantly higher healthy-food choice at post-training), and future research could aim to probe this in more adequately powered studies to determine whether food devaluation plays a role in FSIT effects on children’s food choices. Alternatively, other measures for food-liking could be explored such as a measure of instrumental responding to obtain food items. This outcome has been found to reduce for energy-dense foods after FSIT (Houben and Giesen, 2018), and the measurement task has also been validated in samples of children as young as 4 years old (Savell et al., 2020).

## General Discussion

The studies presented here aimed to explore the effectiveness of different variants of FSIT as a healthy eating tool for primary school aged children. Study 1 found no significant effects of FSIT on food choice behaviour at all. A key difference between this study and positive earlier studies (Folkvord et al., 2016; Porter et al., 2018) was that children participated in groups (mixed by condition) rather than one-on-one. Anecdotally, the group-testing sessions were noisier and more distracting – children would talk during the task despite efforts to keep the room quiet, and they could also turn around and see that their peers were playing a different version of the task than themselves. This is reflected in the data – examining children’s performance data on the emotive-FSIT task (i.e., the only version of FSIT that we had tested beforehand, and with success) showed that commission error rates were unexpectedly high. Children may also have been influenced by each other during the food-choice task itself – some items were clearly very popular, and some children would exclaim in delight upon finding them in the choice task. Children are influenced by the food preferences of their peers (Birch, 1980; DeJesus et al., 2018) and this social endorsement by peers may have overridden FSIT effects on food choices.

In comparison, children in Study 2 participated on a one-on-one basis, as in our own earlier research and that of others (Folkvord et al., 2016). This time, a significant effect of training was observed once more for the FSIT-computer task, which is the same task that has been successfully tested in earlier research. Unlike in Study 1, children’s task performance did not appear to be negatively impacted in this study. This suggests that low commission error rates during FSIT may be important for subsequent training effects on food choices, which dovetails with meta-analyses of studies in adult participants, where it was found that successful stopping on inhibition trials was necessary for FSIT to have an impact on eating behaviour (Jones et al., 2016). To explore this, we conducted an exploratory correlation on the data collected in Study 2, which indicated that changes in commission errors were negatively correlated with changes in healthy-food choice (*R* = −0.223, *p* = 0.009) – in other words, improvements in inhibition to energy-dense foods in the FSIT training tasks were associated with increases in healthy-food choices.

These findings suggest that lower commission error rates lead to stronger FSIT effects on eating behaviour. However, in Study 2, FSIT-computer training appeared to be more effective than FSIT-app training, despite the computer task having significantly higher commission error rates than the app task. This could be due to differences in commission error measurement sensitivity as a result of the response mode (touchscreen taps vs. keyboard press). The computer task left little room for error (i.e., because children’s hands were resting on computer keys, meaning that even very tiny movements can result in a “press”) and was thus a highly-sensitive measure of commission errors. Comparatively, for the app task, the resting position of children’s hands was further away from the response apparatus (it is not possible to play the FSIT-app task with the hand resting on the screen). The greater distance between hand and device may then lead to the recording of artificially low error rates (i.e., because there is more time to correct errors on the hand’s comparatively long journey towards a touch screen). Future research could explore this possibility, and could also investigate whether these task differences impact children’s engagement with FSIT. For example, the increased challenge of computer-based tasks may engage children’s attention and motivation, and compel them to improve their scores and focus on learning the rules of the game. However if the game is less challenging (i.e., because motor responses can be corrected at relative leisure), then there may be less drive to improve performance. The findings of these studies together indicate that such motivation and attention may be key for FSIT effects on eating behaviour.

Altogether, the results of these studies suggest that high task performance is required for FSIT to have an impact on eating behaviour outcomes, and that this may be achieved by implementing training in a controlled and quiet environment. One potential alternative explanation for the difference between studies is that individual testing results in demand characteristics, with children more likely to try and please the experimenter when they are working on a one-on-one basis. In Study 2, we found no significant differences between groups regarding awareness of the study aims, task contingencies, or task effects on food choices/liking. Awareness of the healthy-eating aims and expected task effects were low, although awareness of contingencies within the task was high. Children in the control group who were considered “aware” of the study’s aims and task contingencies described how they needed to press for the “healthy” activity images (sports), and not for the “unhealthy” activity images (technology). This suggests that the control task could also have driven any demand characteristics within the sample, rather than this being limited to the active group only.

However, if children were simply choosing foods based on what they believed the experimenter wanted them to choose, healthy-food selection rates would surely be much higher than they are and similar across all conditions. In reality, very few children chose a high number of healthy foods (and barely any selected a healthy food as their real choice), further suggesting that demand characteristics were not driving these results. Both studies found a decline in healthy eating behaviour across time – this occurred in all groups in Study 1, and in the Control group only in Study 2. Turton et al. (2018) who also observed a decline in the healthiness of participants’ eating behaviour over experimental sessions, suggested that such patterns may be due to participants becoming more familiar with the experimental environment and becoming more relaxed in their eating behaviours. Relatedly, children being offered a snack of their choice in the middle of the school day (Study 2 only) would have been a departure from their usual routine, and may have been seen as a rare chance for them to indulge in a “treat”. In this sense, children may have been in a more disinhibited state than they would normally when choosing which foods to eat. Understanding the wider context of children’s eating behaviours (e.g., whether they had already eaten fruit that day, how often they were allowed energy-dense foods at school and at home etc.) would help to better contextualise these findings.

While the finding of a decline over time departs from previous findings (i.e., Porter et al., 2018 found an increase in healthy-food choice in the FSIT group and no change in healthy-food choice in the control group), this could be due to children in the current study choosing a higher percentage of healthy foods at baseline. An earlier study by our research group (Porter et al., 2018) saw healthy choices rise significantly in the FSIT group from 36 to 52%, whereas in the present study, they were higher at baseline (42–48%) but remained stable to post-training (40–48%). Meanwhile, healthy choices in the earlier study’s two control groups remained stable from baseline (29–36%) to post-training (32–39%) whereas in the present study, baseline choices in the Control group were higher (43%) but then significantly declined to a more comparable 33% at post-training. This suggests that the starting point for children’s food choices could be key for determining whether FSIT has an augmentative effect (i.e., increases healthy-food choice) or a protective effect (i.e., guards against a decline in healthy-food choice); when healthy-food choices are low at baseline then FSIT has the potential to increase them but when healthy-food choices are high at baseline, FSIT can maintain this behaviour.

These studies have a number of strengths; firstly, they provide further support for the use of FSIT as a healthy eating intervention for use with children. While it could be argued that the consistent stimulus-response associations (which are important for FSIT’s efficacy) reinforce potentially harmful and rigid narratives about which foods “should” and “should not” be eaten, it is notable that the effects of FSIT on behaviour are much more subtle than this – after FSIT children choose a slightly higher number of healthy foods (Porter et al., 2018) and consume a slightly smaller amount of energy-dense foods (Folkvord et al., 2016), however, they do not completely stop choosing or eating these foods. Similarly, work with adults has shown that FSIT leads to subtle reductions in liking of energy-dense foods (e.g., Veling et al., 2017b), which could help people achieve a more balanced diet without needing to entirely cut out their favourite energy-dense foods. A further strength is that Study 2 also piloted a FSIT app with children for the first time and provides preliminary, tentative evidence that this app may be able to support healthy eating habits in children (i.e., by protecting against the observed decline in healthy behaviours over time). As FSIT can be delivered as a DBCI directly to users’ devices (such as *via* the FoodT app), this intervention can be used immediately and for free by families. A further advantage is that the flexibility that DBCIs afford users means that recommendations for usage based on the findings of this study (i.e., to preferably play the app in a quiet environment) can be implemented in a way that suits them. The smaller effect size for this app (in comparison to computer-based FSIT) suggests that further research needs to be conducted to identify the reasons for this, and potential developments to optimise app-based training should be identified. A further strength of this study is that a food-liking rating scale was successfully piloted which could be used in future research to pursue the question of whether the stimulus devaluation contributes to FSIT effects on children’s eating behaviour as well as adults’.

Nevertheless, a number of limitations should also be noted. Most notably, the question of whether evaluative conditioning can bring additional benefits to FSIT paradigms has not been fully answered. In Study 1 (in which we could directly compare neutral and emotive No-Go signals), no training effects were observed. In Study 2 (in which training effects were observed), the two FSIT tasks differed in a number of ways beyond the response signals used, and therefore the relative contribution of these various factors cannot be teased apart. For example, a further potentially crucial difference between the app and computer tasks is the proportion of critical “food-response” trials per block – in the app this comes to 50% of all trials (plus 50% “filler” trials) whereas in the FSIT-computer task, 100% of trials encouraged a food-response association. Therefore, the level of exposure to stimulus-response associations was lower in the FSIT-app group compared to the FSIT-computer group, which may have impacted the efficacy of this task variant. Earlier research with children (Folkvord et al., 2016; Porter et al., 2018) has found significant, positive effects of FSIT using tasks that do not contain these fillers, suggesting that simpler tasks with a higher proportion of food-response trials may be most effective for children. Future research should aim to test the influence of these various factors (including the use of emotive vs. neutral response signals) in tasks that more closely control for other differences. A second limitation is that the researcher who delivered the intervention, recorded the outcome measures and performed the statistical analysis was not blinded to condition allocation. Finally, current results do not help to answer the question of how long any FSIT effects on food choices might last for, and whether effects can be reinforced by repeated training sessions. A more longitudinal design, such as that used by Study 1, would help to explore this question.

Future research should aim to investigate whether repeated use of FSIT at home can have a significant impact on real-life eating behaviour, as has been found to be the case with adult participants. While the outcome measures used here are useful for gathering preliminary evidence on FSIT effects within a controlled environment, their ecological validity is questionable. For example, the hypothetical food-choice task (when implemented as in Study 2) does not allow children to change their choices after they have made their initial selections. It is questionable whether this is truly representative of children’s daily feeding decisions compared to tasks in which they are allowed (at least some) time to deliberate over their choice and select an alternative if they change their minds. Work with adults has suggested that the effect of response training paradigms may be limited to choices made under time-pressure. While this could be a further explanation for the lack of effects in the real-food-choice task, it also has clear implications for the applied value of this paradigm as a healthy eating intervention. Folkvord et al. (2016) found an effect of FSIT on calorie intake without time pressure, however, no studies have yet investigated the impacts of FSIT on children’s real life eating behaviour outside of an experimental setting.

## Conclusion

To conclude, the studies presented here provide some further support for the efficacy of FSIT as a healthy eating tool for children. Accuracy on energy-dense food No-Go trials appears to be important for FSIT effects on eating behaviour, and conditions that reduce children’s attention or motivation (such as noisy, distracting environments) may subsequently reduce training effects on food choices. Future research should explore whether app-based versions of FSIT can be optimised (i.e., by increasing the level of challenge) to increase the efficacy of this delivery mode, and whether FSIT effects on food choices can translate into real life eating behaviour over longer time periods.

## Data Availability Statement

The research data and analysis code supporting this publication are openly available from the University of Exeter’s institutional repository at: https://doi.org/10.24378/exe.3303.

## Ethics Statement

The studies involving human participants were reviewed and approved by University of Exeter CLES Psychology Ethics Committee. Written informed consent to participate in this study was provided by the participants’ legal guardian/next of kin.

## Author Contributions

LP: conceptualisation, methodology, software, investigation, data curation, formal analysis, and writing. FG: conceptualisation, writing (review and editing), and supervision. KW: conceptualisation (Study 2), writing (review and editing), and supervision (Study 2). FV: conceptualisation (Study 1), methodology (Study 1), writing (review and editing), and supervision (Study 1). NL: conceptualisation, methodology, formal analysis, writing (review and editing), and supervision. All authors contributed to the article and approved the submitted version.

### Conflict of Interest

The authors declare that the research was conducted in the absence of any commercial or financial relationships that could be construed as a potential conflict of interest.

## Footnotes

^1^http://www.exeter.ac.uk/foodt

^2^For pragmatic reasons associated with access to university laptops with EPrime software, a further (harder) variant of the task was developed using an online server and tested simultaneously in a separate sample of children. The results regarding this variant are not reported here but will appear in the lead author’s upcoming thesis.

^3^https://get-information-schools.service.gov.uk/ and https://www.devon.gov.uk/factsandfigures

^4^This differs from the procedure in Porter et al. (2018) where children were told they would be given one of their choices to motivate ecologically valid choices. This was not possible in the current study due to group-testing, and the logistical issues involved in transporting required amounts of equipment and food *via* public transport.

^5^http://www.lifesci.sussex.ac.uk/home/Zoltan_Dienes/inference/Bayes.htm

^6^A meta-analysis of studies performed by our research group with child participants yielded a medium effect size of *d* = 0.446, which translates as *f* = 0.223. Some of the studies included in this meta-analysis involved group-testing studies, and as noted in Study 1, it was observed that group-work studies produced smaller effect sizes than individual-testing studies. As the current study used an individual-testing methodology, the standard medium effect size of *f* = 0.25 was used as a closer estimate of the true effect size for this method type.

^7^Resources consulted = https://get-information-schools.service.gov.uk/ and https://data.london.gov.uk/dataset/ethnic-groups-borough.
